# Immunohistochemical and Molecular Characterization of the Human Periosteum

**DOI:** 10.1155/2013/341078

**Published:** 2013-05-02

**Authors:** Sönke Percy Frey, Hendrik Jansen, Stefanie Doht, Luis Filgueira, Rene Zellweger

**Affiliations:** ^1^Department of Trauma, Hand, Plastic and Reconstructive Surgery, University of Würzburg, Oberdürrbacher Street 6, 97080 Würzburg, Germany; ^2^Department of Anatomy and Human Biology, University of Western Australia, Perth, WA 6009, Australia; ^3^Department of Orthopedic and Trauma Surgery, University of Western Australia, Perth, WA 6000, Australia

## Abstract

*Purpose*. The aim of the present study was to characterize the cell of the human periosteum using immunohistological and molecular methods. *Methods*. Phenotypic properties and the distribution of the cells within the different layers were investigated with immunohistochemical staining techniques and RT-PCR, focussing on markers for stromal stem cells, osteoblasts, osteoclasts and immune cells. *Results*. Immunohistochemical results revealed that all stained cells were located in the cambium layer and that most cells were positive for vimentin. The majority of cells consisted of stromal stem cells and osteoblastic precursor cells. The density increased towards the deeper layers of the cambium. In addition, cells positive for markers of the osteoblast, chondrocyte, and osteoclast lineages were found. Interestingly, there were MHC class II-expressing immune cells suggesting the presence of dendritic cells. Using lineage-specific primer pairs RT-PCR confirmed the immunofluorescence microscopy results, supporting that human periosteum serves as a reservoir of stromal stem cells, as well as cells of the osteoblastic, and the chondroblastic lineage, osteoclasts, and dendritic cells. *Conclusion*. Our work elucidates the role of periosteum as a source of cells with a high regenerative capacity. Undifferentiated stromal stem cells as well as osteoblastic precursor cells are dominating in the cambium layer. A new outlook is given towards an immune response coming from the periosteum as MHC II positive immune cells were detected.

## 1. Introduction

Periosteum is a bilayered membrane associated with bone. It is located at the boundary between the surrounding soft tissues and the cortical bone to which it is anchored through Sharpey's fibers. It forms a fibrovascular membrane covering the external surface of the bone, except for any articular cartilage, ligament, or tendon insertions. The periosteal membrane consists of an outer fibrous layer containing the blood vessels and nerves, which supply the bone, and an inner osteogenic (cambium) layer, which lies adjacent to the bone. The cells of the cambium layer are embedded in a loose connective tissue and are surrounded by a dense microcirculation. Cells of the cambium layer have been shown to contribute to fracture healing in animal models [1]. Generally, periosteal cells are important for fracture healing and bone remodeling [2], and in children, the cambium layer contributes to external appositional bone growth. In both children and adults, the periosteum provides a pool of progenitor cells endowed with the potential to differentiate into an osteochondrogenic cell lineage as part of the fracture healing process [3, 4]. However, in elderly people, the periosteum diminishes in thickness and osteogenic potential. Periosteal cells show high mitogenic activity immediately adjacent to the site of injury prior to the initiation of osteochondral differentiation [5]. Cambium cells may be activated after mechanical stimulation, such as soft tissue trauma [6], by infection or by some tumors [4]. Under these circumstances, this layer is capable of inducing callus tissue formation and osteogenesis. Ito et al. [7] suggest that the cambium layer serves as a reservoir of undifferentiated cells that are able to differentiate into lineages of both osteoblasts and chondrocytes. The ability of cambium cells to differentiate into chondrocytes has stimulated more recent interest in using periosteal grafts for the resurfacing of full thickness articular cartilage defects. Therefore, periosteal autografts have been used as flaps with the cambium cell layer facing towards the defect the articular cartilage [8]. In addition, periosteum has also been described as being able to induce heterotopic bone formation when implanted into muscle as a free graft.

There is a wide range for the use of periosteum in clinics. It can be used in stimulating growth in the shorter limb in patients, for example, with lower limb discrepancy via periosteal stripping [9] or as a promising cellular candidate for bone tissue engineering [10]. Considerable attention has been focused on its rich osteogenic properties and its potential as a tissue engineering material for use in the repair of bone defects. 

Despite the paramount importance of knowing the properties and distribution of periosteal progenitor cells, little has been published on the characterization and quantification of human periosteal stem cells. Consequently, the present study aimed to characterize human periosteal cells using immunohistologicaland molecular methods by investigating phenotypic properties and the distribution of osteogenic stem cells in the different layers of periosteum. Of particular interest was the identification of osteoblast, chondrocyte, and osteoclast lineages.

## 2. Methods

### 2.1. Periosteum Samples

A total of 15 periosteal explants (10  ×  5 mm^2^) were taken from the medial aspect of proximal tibia of patients who underwent orthopedic surgical procedures at the proximal tibia. Seven samples were stained by immunohistochemistry, and other eight samples were processed for reverse transcription polymerase chain reaction (rt-PCR). The age of the tissue donors varied between 18 and 62. The study group included eight males and seven females. Research approval and ethics clearance were acquired from the Royal Perth Hospital Human Research Ethics Committee, and, written informed consent was obtained from all study participants.

Selection criteria for periosteal tissue donation included intact periosteum; absence of any severe medical condition; and being aged between 18 and 65 years. Postharvest exclusion criteria included tissue damage during harvesting, with loss of one of the layers; absence of a clear distinction between the fibrous and cambium layer; artifacts (folding, tearing, dehydration, cleavage, and overstaining); and improper orientation during sectioning.

### 2.2. Fluorescence Microscopy

**Figure 1:**
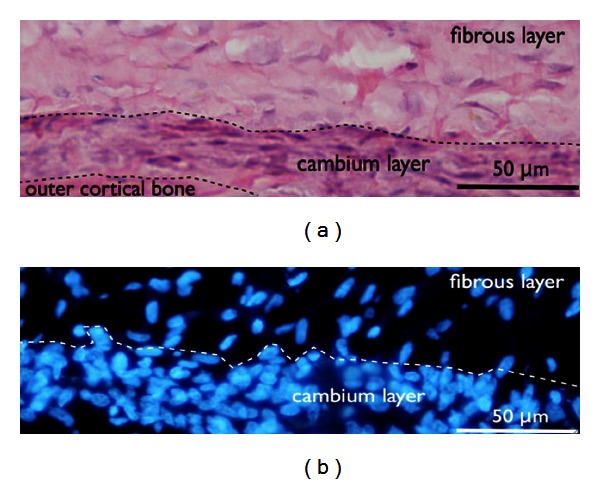
(a) Conventional light microscopy. Hematoxylin and eosin-stained periosteum samples showing the cell rich cambium layer and the upper fibrous layer. (b) Fluorescence microscopy demonstrating a DAPI stain showing the distribution of nuclei (blue).

The fresh periosteum was carefully removed from the underlying bone using blunt forceps in order to keep the fibrous and the cambium layers intact. In some cases, a portion of underlying bone and overlying muscle tissue was also extracted. The periosteal tissue was immediately fixed in 1% paraformaldehyde in 0.1 M sodium phosphate buffer (pH  7.2) for at least 4 hours. The samples were subsequently frozen in liquid nitrogen and mounted vertically into OCT compound (Tissue-Tek no. 4583, Thuringowa, QLD, Australia) for cryosectioning and in order to make sure that the entire thickness of the periosteum was included in the sections. Cryosectioning was done with a Leica Cryostat CM 30505 (Leica Microsystems Nussloch GmbH, Nussloch, Germany). 8 *μ*m thick cross-sections were cut at a temperature of −15°C. The first sections were stained with hematoxylin/eosin to check the orientation of the periosteum and to ensure that both the cambium and the fibrous layer were completely included (for representative example see Figure 1). 

For immune staining, the samples were incubated with 0.2% Triton X-100 in phosphate buffer saline (PBS) for 10 minutes at room temperature. For (tartrate-resistant acidic phosphatase stain TRAP, the cells were processed according to a fluorescence-based protocol [11, 12]. Briefly, the cells were incubated for 15 min with ELF97 substrate (20 *μ*M, E6569; Molecular Probes) in 110 mM acetate buffer (pH  5.2) containing 1.1 mM sodium nitrite and 7.4 mM tartrate (Sigma Aldrich). The nuclei were stained with DAPI (4′,6-diamidine-2′-phenylindole dihydrochloride, 10 ng/mL; Roche Diagnostics, Mannheim, Germany). 

Antibodies against the following epitopes were used for immune staining: Stro-1 (mAB mouse IgGI, hybridoma supernatant, Developmental Studies Hybridoma Bank at the University of Iowa), alkaline phosphatase (ALP: hybridoma cell supernatant clone B4-78, Developmental Studies Hybridoma Bank at the University of Iowa), vimentin (mAB mouse VIM-13.2 clone, mouse ascites fluid, Sigma), MHC class II (Dako, Glostrup, Denmark), CD3 (CB3G clone, Hybridoma supernatant, 1 : 100 dilution, Eppler et al. 1996), and core binding factor alpha-1 (Cbfa-1)/runt-related transcription factor 2 (Runx2) (1 : 100 dilution, rabbit polyclonal antibody, Alpha Diagnostic Intl. Inc.). The following secondary antibodies were used: Donkey anti-mouse AlexaFluor 488, Donkey anti-mouse AlexaFluor 555, Donkey anti-rabbit AlexaFluor 488 and Streptavidin AlexaFluor 546 (all from Invitrogen/Molecular Probes). All steps were performed at room temperature. Finally, the stained sections were mounted in fluorescent mounting medium (Dako). Fluorescent samples were analyzed and documented using confocal microscopy (Bio-Rad MRC 1024, Coherent Enterprise argon ion 250-mW multiline UV, American laser 100-mW argon ion multiline laser, and Melles Griot 0.5-mW green helium-neon laser; Bio-Rad, Hercules, CA, USA). Conventionally stained samples were documented and analyzed with Nikon Eclipse 90 I (Nikon Corporation, Kawasaki, Japan) and Nikon Digital Eclipse DXM using ACT-1 software 1200 (Nikon Corporation, Kawasaki, Japan). For all stainings, negative controls were included without the first antibody. No unspecific staining was detected for the antibodies and concentrations used in this study. 

### 2.3. Real-Time Reverse Transcription Polymerase-Chain Reaction

After excision and making sure that no bone or muscle tissue was included, the periosteal tissue was immediately dissected into 1 mm pieces with a surgical knife. This was immediately followed by extraction of total RNA using the Ultraspec TM-II RNA Isolation system (Biotecx Laboratories Inc., Houston, TX, USA) in accordance with product guidelines. One microgram of total RNA was reverse transcribed to complementary DNA (cDNA) using Promega Reverse Transcription System (Promega Corporation, WI, USA). Real-time quantitative PCR was performed using the iQ SYBR Green Supermix (Bio-Rad, Regents Park, NSW, Australia) and the Rotor-Gene 3000 cycler and corresponding software (Corbett Life Science, Mortlake, NSW, Australia). Using gene-specific primers (see Table 1) for human cDNA, 1 *μ*g of cDNA was amplified for the following genes: alkaline phosphatase, Cbfa-1/Runx2, RANK-L, M-CSF, SP7-s (short splice variant of Osterix), SP7-l (long splice variant of Osterix), Sox-9, TRAP, calcitonin receptor (CTR), cathepsin K, and *β*-actin (positive control). The fluorescence profile during amplification was controlled, and only samples in which the signal remained at baseline level during early cycles, increased exponentially, and finally reached a plateau phase at high-cycle numbers, were used. In addition the quality of PCR products were tested by including the melting/dissociation curve and detection of corresponding molecular product weight on agarose gels. Only results complying with this quality control were included.

## 3. Results

### 3.1. Characterization and Quantification of Cells by Immunofluorescence

**Figure 2:**
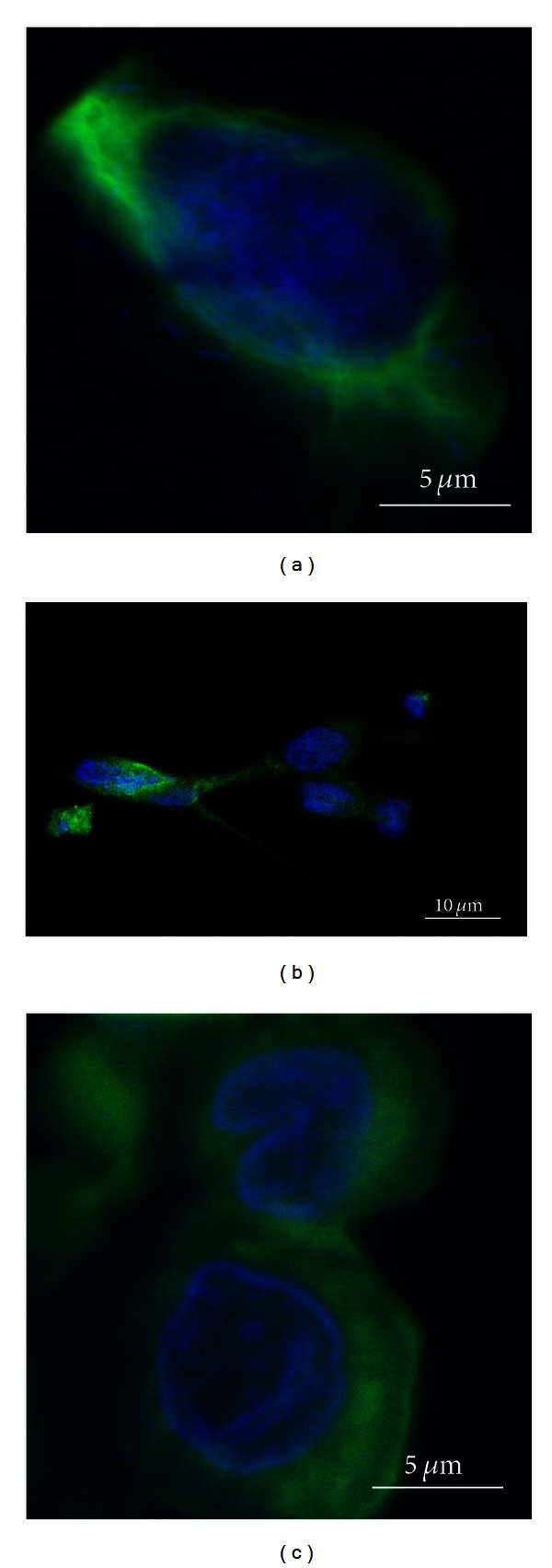
Confocal fluorescence microscopy (a–c) of representative cells located in the cambium layer and stained for markers of the osteoblast lineage. Nuclei were stained with DAPI (blue). (a) Stromal cells stained positive for vimentin (green). (b) Stromal stem cells stained positive for Stro-1(green). (c) Cells of the osteoblast lineage stained positive for Cbfa-1/Runx2 (green).

**Figure 3:**
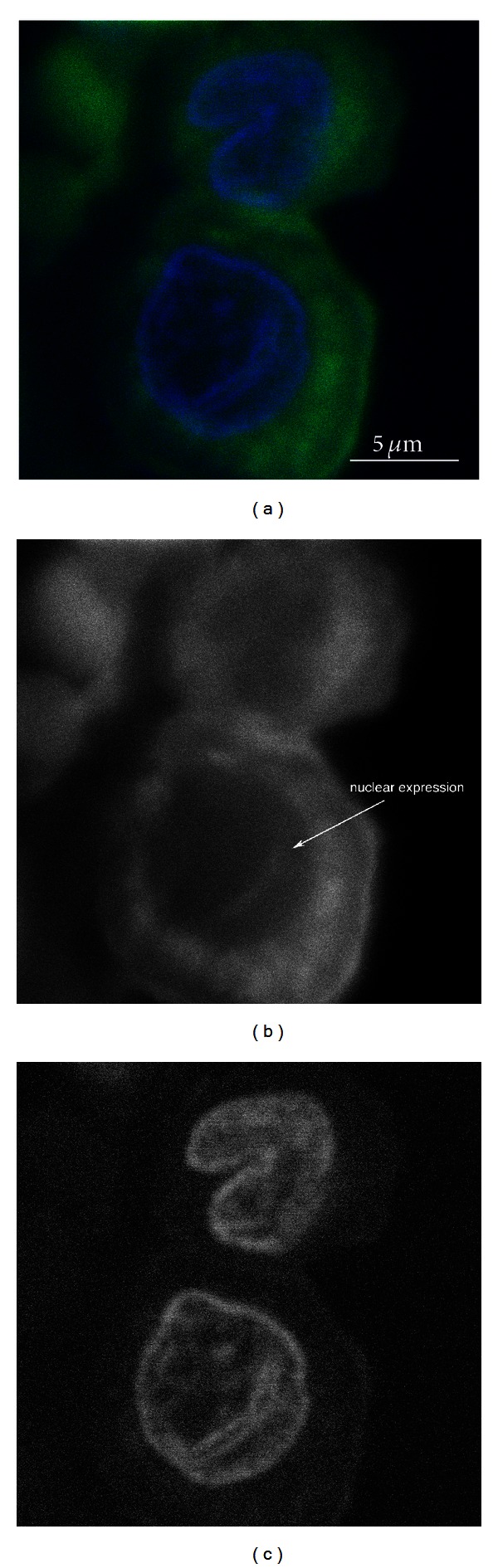
Confocal fluorescence microscopy of cells located in the cambium layer and stained for Cbfa-1/Runx2. (a) Merged fluorescence image showing in blue the nuclear DAPI stain and in green the staining for Cbfa-1/Runx2. (b) Cbfa-1/Runx2 staining in black and white, note the nuclear expression pattern (arrow) in addition to the distinct cytoplasmic expression pattern. (c) Nuclear stain only in black and white.

**Figure 4:**
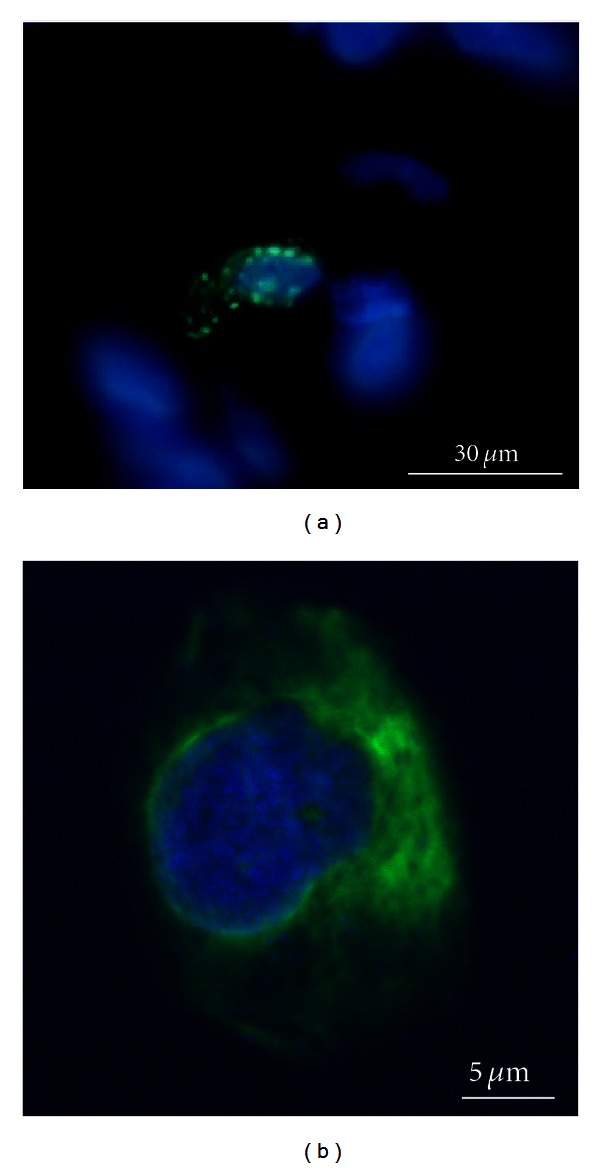
Confocal fluorescence microscopy of a representative osteoclast-like cell positive for TRAP (a) and an immature dendritic cell positive for intracellular MHC class II (b).

Cryosections were first stained for conventional light microscopy with hematoxylin and eosin (a) in order to make sure that both periosteal layers were included on the section (Figure 1). In comparison, just the nuclei of the two different layers were seen on the DAPI stain (b). Subsequently, the sections were processed with specific stains for fluorescence microscopy. The samples were then analyzed using confocal microscopy. The fibrous layer, which stabilizes the periosteum, consisted largely of a dense, cell poor connective tissue. The cells had a fibroblastic spindle-like shape and were of uniform size. There was no specific staining of cells in the fibrous layer for the markers used. In contrast, the immunohistochemical evaluation of the cambium layer, which is the interface between the periosteal fibrous layer and the outer cortical bone, revealed a cell rich tissue with a variety of different cell types (Figures 2–4). 

### 3.2. Reverse Transcriptase Polymerase Chain Reaction (RT-PCR)

Periosteal samples were tested for additional markers using real-time RT-PCR due to the lack of antibodies specific for certain markers and also in order to confirm the histological results. We were interested in identifying the presence of osteoblast-, osteoclast-, and chondrocyte-related cell populations. Markers of osteoblasts included M-CSF, Cbfa-1/Runx2, SP7/Osterix (long and short splice variants), RANK-L, and ALP. Sox-9 was used as a chondrocyte marker. TRAP, cathepsin K, and CTR were used as osteoclast markers (Table 1).

M-CSF and CBFA-1/Runx2 were highly expressed. Rank-L was expressed almost as highly. Expression of SP7-s and ALP was detected at lower amounts than the other osteoblastic markers. There was no detection of SP7-l. The finding of mRNA expression of Cbfa-1/Runx2, Osterix, ALP, M-CSF and RANK-L indicated that there were cells of the osteoblast lineage in the periosteum. Interestingly, the mRNA for the transcription factor Sox-9 was also detected. This is a marker for chondroprogenitor cells and a nuclear transcription factor that plays an essential role in chondrogenesis. All osteoclastic markers including calcitonin receptor, TRAP, and cathepsin K were expressed, confirming the presence of osteoclasts or their late precursors in the periosteum. 

In summary, we can say that RT-PCR results (summarized in Table 2) confirmed the immunofluorescence findings. Stromal stem cells as well as osteoblastic cells were stained positively using specific markers (Stro-1, Cbfa-1/Runx2, vimentin). The expression of osteoblastic markers (CBFA-1/Runx2, ALP, SP7/Osterix, M-CSF, and RANK-L) supports the immunohistochemical findings. Immunofluorescence microscopy also revealed the existence of TRAP and MHC class II positive cells, suggesting the presence of osteoclasts and dendritic cells, respectively. Positive findings of transcripts of osteoclastic markers (CTR, TRAP, and cathepsin K) as well as of osteoclast differentiation factors that are expressed by osteoblasts (M-CSF, RANK-L) emphasize the presence of osteoclasts. 

## 4. Discussion

Open fractures but also bone tumors are just one of the samples bone defects can arise from. Current knowledge suggests that the periosteum, a fibrous tissue which covers the surface of all bones, contains a population of progenitor cells which mediate the repair of bone defects. Exogenous growth factors or allografts are often used to bridge bone defects. Over the last years many efforts have been made in order to identify natural sources of osteogenic cells for the success of bone bioengineering. Among them, periosteum tissue has emerged as an interesting candidate. Its cambium layer offers a biological reservoir of stromal stem. The multipotency and growth characteristic of periosteum-derived progenitor cells is known [13]. Therefore, isolated single periosteal cells or freshly harvested periosteum are known to have chondrogenic and osteogenic potential and are popular materials for tissue engineering. Harvested periosteum tissue itself can also be used as a natural scaffold in a variety of clinical applications such as repair of cartilage defects [14] or enhancement of soft tissue fixation to bone [15]. Therefore, flaps of periosteal autografts have been frequently used for the repair of articular cartilage [16]. In the management of recalcitrant nonunions with small gaps, both periosteal and corticoperiosteal flaps play a central role [17]. To bridge segmental bone defects Li et al. implanted Cbfa-1 gene-modified tissue-engineered bone and vascularized periosteum. Herein, defects were stimulated by osteogenesis, osteoinduction, and osteoconduction [18]. Even in the treatment of postradiation-induced fractures or chronic nonunions with poor chances of spontaneous healing and a concomitant small skin defect, the transfer of free combined vascularized corticoperiosteal-cutaneous flaps seems to be ideally suited [19]. Free bone or periosteal flaps from the medial femoral condyle are being employed for treatment of recalcitrant nonunions. When harvested in a corticocancellous fashion, these flaps have the potential to compromise the stability of the femur [20]. Periosteal grafts provide successful reconstruction of skeletal problems of the distal radius, wrist, and hand [21]. Previous studies showed that periosteum and endosteum are major local sources for skeletal progenitors with distinct osteogenic and chondrogenic potentials [22]. The osteogenic potential of periosteum has been investigated in several models. In an experimental rabbit study, heterotopic ossification was explored. Herein, periosteum was elevated as a vascularized flap and showed strong osteogenic capacity [23]. As Castro-Silva et al. describe periosteal-derived cells present an interesting potential to differentiate in mature osteoblasts able to promote mineralization in vitro by incorporating to ECM circulating calcium from extracellular compartment [24]. Stro-1 positive cells as detected in our samples may undergo proliferation and differentiation into chondroblasts and osteoblasts [25]. More than half of the detected cells were positive for stro-1 demonstrating the presence of stromal stem cells particularly in the deep layer of the cambium indicating that the periosteum contains its own residential stem cell population. Besides, we noticed, coming closer to the cortical bone, that the number of stro-1 positive cells increased the proposing of the cell layer responsible for the regenerative capacity of the periosteum which lies directly on the cortical side of the bone (deeper cambium layer). Consequently, the fact of having Stro-1 positive stromal stem cells in the cambium layer offers an explanation as to why periosteum maintains the potential of inducing its cells to differentiate into a variety of lineages including osteoblasts and chondrocytes [1, 4, 7, 26].

As interesting as the fact that these cells might stimulate bone growth, they also potently modulate immune responses, exhibit healing capacities, improve angiogenesis, and prevent fibrosis [26]. Most interestingly, in regard to immune response modulation, we also found scattered MHC II expressing cells in the cambium layer of the periosteum which was not described before. These MHC II positive cells are most likely immature dendritic cells, collecting antigens in this tissue before migrating towards the regional lymph nodes [27]. As none of the patients had an infection or a tumor, cells positive for MHC II were infrequent which presumes that periosteum is one organ in the chain of the immune response. During an inflammatory process, cells might be multiplied which explains why the average of MHC II positive cells differed among the tissue samples.

Matrix resorbing cells play an important role in the removal of bone debris after injury and in extracellular matrix remodeling [28]. According to Fan et al. the number of osteoclasts is higher coming along with a thicker cambium layer in osteoporotic rats [29]. In our study the presence of osteoclasts or their precursors was detected with molecular and morphological methods. Expression of mRNA for additional osteoclast markers CTR and cathepsin K was also found. TRAP positive cells were mainly found in the deeper layer of the cambium layer close to the outer cortical bone which affirms the findings of Fan et al..

Taken together, it is interesting that there are both dendritic cells and osteoclasts located in the same tissue, as both cell types derive from the same myeloid precursors [30–32]. Either both cell types have already committed themselves towards one or the other sublineage before entering the tissue or there are separate niches for the differentiation of the two functionally different cell types, especially as there were no TRAP and MHC II double positive cells detected in our samples. RANK-L is an activating factor for dendritic cells [33] as well as for osteoclasts. In contrast, Miyamoto et al. [32] suggest that addition of M-CSF inhibits this maturational step for dendritic cells potently modulating immune responses. M-CSF and RANK-L, expressed on osteoblasts, provide the differentiation and survival signal of these cells [3, 27, 34]. Both M-CSF and RANK-L have been shown to be expressed in the periosteum in this study, indicating that there are functional osteoblast-like cells recruiting and interacting with osteoclast precursors. Finally, both cell types were detected in lower numbers than stromal stem cells or osteoblastic precursor cells indicating a higher capacity towards regeneration. Besides, these cells might be a novel target to be addressed in the treatment of osteoarthritis, rheumatoid arthritis, genetic bone, and cartilage disorders as well as bone metastasis. But also bone healing commences with an inflammatory reaction, which initiates the regenerative healing process leading in the end to reconstitution of bone. An unbalanced immune reaction during this early bone healing phase is hypothesized to disturb the healing cascade in a way that delays bone healing and jeopardizes the successful healing outcome.

Furthermore, microscopic detection of Cbfa-1/Runx2+ cells in the cambium layer and detection of mRNA expression for osteoblastic markers (Cbfa-1/Runx2, M-CSF, RANK-L, Osterix, and ALP) indicate that there are cells already committed to the osteoblast lineage. Differences in expression levels for the different osteoblastic markers may indicate a variety of cells at different stages of differentiation. Especially, Cbfa-1/Runx2 is a prerequisite of osteoblast differentiation. Enzyme activity of ALP is believed to be one of the major characteristics of osteoblasts [35]. This may be similar to Stro-1 positive human marrow stromal cells. Ahdjoudj et al. [36] found clonal expressed mRNA markers, or protein, of the osteoblast lineage, the chondrocyte lineage, and even the adipocyte lineage, suggesting a common precursor stage for the three lineages. Depending on close surrounding environment and the influence of specific transcription factors, the precursor cells may differentiate into osteoblasts, chondrocytes, or adipocytes [37, 38]. In addition, it explains why periosteum is so important for bone fracture healing and callus formation, as Stro-1 positive cells may undergo proliferation and differentiation into chondroblasts and osteoblasts [25].

Osterix plays a crucial role in segregating the osteoblast and chondrocyte lineages from bipotential osteochondroprogenitor cells during bone formation. Having detected expression of Osterix in this study indicates that there are cells committed to the osteoblastic lineage in the cambium layer. Osterix fulfills its function by inhibiting Sox-9 expression and by fully establishing the osteoblast phenotype [39]. Nonetheless, the nuclear transcription factor Sox-9, which has been reported to be expressed in all chondroprogenitors and which has an essential role in chondrogenesis [40], was found to be expressed in human periosteum in our study. This underlines that there are progenitor cells with the potential of becoming chondrocytes in the cambium layer. In contrast to our findings, Ball et al. did not find a positive marker for mature chondrocytes [41]. 

The present study characterized and quantified human periosteal cells with immunohistochemical and molecular methods. Cells of the osteoblastic and the chondroblastic lineage, as well as their precursor stages, were detected in the cambium layer of the human periosteum. In addition, osteoclasts and dendritic cells were also localized in the same layer. Hopefully, this study provides further incentive for future investigation of the function and interplay of human periosteal cells. In sum, our work elucidates the role of periosteum as a source of cells with a high regenerative capacity. Undifferentiated stromal stem cells as well as osteoblastic precursor cells are dominating in the cambium layer. A new outlook is given towards an immune response coming from the periosteum as MHC II positive immune cells were detected. Instead of using exogenous growth factors and in order to advance therapeutic protocols to solve posttraumatic or degenerative bone lesions, periosteum should be explored more intense as an available biological answer.

## Figures and Tables

**Table 1:** Specific primers used for RT-PCR.

Gene	Annealing temperature	Application of primer	Forward and reverse primers (5′→3′)
ALP	50.0°C	Forward	ACG TGG CTA AGA ATG TCA
	51.0°C	Reverse	CTG GTA GGC GAT GTC CTT A
Cbfa-1/Runx2	48.0°C	Forward	TCT TCA CAA ATC CTC CCC
	48.0°C	Reverse	TGG ATT AAA AGG ACT TGG
RANK-L	58.0°C	Forward	CCT ACG CAC AAG GCG AAG ATG C
	58.0°C	Reverse	CGT AGA CCA CGA TGA TGT CGC C
M-CSF	50.0°C	Forward	CTG ACC AGC TCA GAG AGA
	44.0°C	Reverse	CTC ATC AAT GTG CAG GA
SP7-s	55.0°C	Forward	CAG GTT CCC CCA GGA GGA
	58.0°C	Reverse	AGT CCC GCA GAG GGC TAG AG
SP7-l	56.0°C	Forward	TCC TCC CTG CTT GAG GAG GA
	58.0°C	Reverse	AGT CCC GCA GAG GGC TAG AG
Sox-9	57.0°C	Forward	GCC ACG GAG CAG ACG CAC
	55.0°C	Reverse	GCG CCT GCT GCT TGG ACA
TRAP	60.0°C	Forward	CTG GCT GAT GGT GCC ACC CCT G
	60.0°C	Reverse	CTC TCA GGC TGC AGG CTG AGG
CTR	53.0°C	Forward	GCA ATG CTT TCA CTC CTG AGA AA
	53.0°C	Reverse	AGT GCA TCA CGT AAT CAT ATA TC
Cathepsin K	50.0°C	Forward	CCC GAA GGG AAA CAA GCA
	50.0°C	Reverse	GCC TGT ACC TGT ACA GCA
*β*-Actin	52.0°C	Forward	GGGTCCGTTTGCCAGTCA
	44.0°C	Reverse	ATCCTCGTCGTACACGTA

**Table 2:** Quantitative Rt-PCR.

Primer	Expression level
ALP	++
Cbfa-1/Runx2	+++
RANK-L	++
M-CSF	+++
SP7-s	++
SP7-l	−
TRAP	+
CTR	++
Cathepsin K	++
SOX-9	+

## Abbreviations

ALP: Alkaline phosphatase
Cbfa-1: Core-Binding factor, alpha 1
Runx2: Runt-related transcription factor 2
RANK: Receptor activator of NF-*κ*B
RANK-L: RANK ligand
M-CSF: Macrophage colony-stimulation factor
SP7/Osx: Osterix
SOX-9: SRY (sex-determining region Y) box 9
TRAP: Tartrate-resistant acid phosphatase
CTR: Calcitonin receptor.
